# Complete Genome Sequence of Staphylococcus aureus Myophage Maine

**DOI:** 10.1128/MRA.01050-19

**Published:** 2019-10-03

**Authors:** Russell Moreland, Abby Korn, Heather Newkirk, Mei Liu, Jason J. Gill, Jesse Cahill, Jolene Ramsey

**Affiliations:** a Center for Phage Technology, Texas A&M University, College Station, Texas, USA; Queens College

## Abstract

Multidrug-resistant strains of Staphylococcus aureus cause serious human disease worldwide. Bacteriophages offer a promising alternative to traditional antibiotics. Here, we announce the 141,712-bp genome of S. aureus phage Maine. A myophage with 9,019-bp predicted terminal repeats and high similarity to other Staphylococcus phages, Maine falls into the Twort-like group.

## ANNOUNCEMENT

Staphylococcus aureus is a Gram-positive member of the human flora. As an opportunistic pathogen, it is a serious public health threat due to the rise of infections by methicillin- and/or vancomycin-resistant strains, as named since 2013 by the Centers for Disease Control and Prevention (https://www.cdc.gov/drugresistance/biggest_threats.html). Bacteriophages are a potential alternative therapeutic against these pathogens (1). Here, we present the complete genome sequence of S. aureus phage Maine.

Bacteriophage Maine was isolated from swine skin samples (Fort Worth, TX) based on its ability to grow on S. aureus strain NRS384 (also known as USA300-0114; see Acknowledgments). The skin swab gauze pads were eluted in Trypticase soy broth (TSB) medium (Difco) at room temperature with shaking for 2 h and then centrifuged at 8,000 × *g* at 4°C for 10 min, followed by passage through a 0.2-μm syringe filter. The phage and host propagations were carried out aerobically at 30°C in TSB medium/agar (Difco), using the soft-agar overlay method (2). Morphology was determined from phage samples stained with 2% (wt/vol) uranyl acetate and observed by transmission electron microscopy at the Texas A&M University Microscopy and Imaging Center (3). Genomic DNA was purified from the phage as described by Summer (shotgun library modification) for the generation of libraries with the Illumina TruSeq Nano low-throughput kit (4). Sequencing of phage DNA was performed by an Illumina MiSeq 250-bp paired-end run with v2 500-cycle chemistry. A total of 400,741 reads from the phage-containing index were inspected with FastQC (http://www.bioinformatics.babraham.ac.uk/projects/fastqc/), trimmed with the FastX Toolkit v0.0.14 (http://hannonlab.cshl.edu/fastx_toolkit/), and then assembled into a single contig with 102-fold coverage using SPAdes v3.5.0 at default parameters (5). Confirmation that the contig was complete came from Sanger sequencing of PCR products amplified off the genome ends (forward, 5′-AGAGGTGATTCTAGTTGGATAAGGAG-3′; reverse, 5′-ACTTGACAAAGGTATCAGTATATGC-3′). All annotation was done using the Center for Phage Technology’s Web Apollo instance after analysis in Galaxy (https://cpt.tamu.edu/galaxy-public/) (6, 7). Unless otherwise specified, all tools were executed with default parameters. First, genes were called from ARAGORN v2.36, GLIMMER v3.0, and MetaGeneAnnotator v1.0 outputs (8–10). Terminators that are rho independent were predicted with TransTermHP v2.09 (11). Functional assignment relied primarily on BLAST searches with a 0.001 maximum expectation value against the NCBI nonredundant and UniProtKB Swiss-Prot/TrEMBL databases (12, 13). Additionally, InterProScan v.5.22-61 conserved domain searching, coupled with TMHMM v2.0 results, was also used (14, 15).

Maine has a 141,712-bp genome with a 30.4% G+C content, similar to the ∼33% G+C content of its host (16). This myophage encodes 219 predicted coding sequences, 59 with predicted functions, and 4 tRNA genes, for a 90.1% coding density. As determined by progressiveMauve, Maine shows 83.22% and 93.18% nucleotide sequence identity across the genome to the other Staphylococcus phages K (GenBank accession number KF766114) and JD007 (GenBank accession number JX878671), respectively (17). PhageTerm predicts that Maine has 9,019-bp terminal repeats (18). Maine therefore appears to belong to the Twort-like group of myophages.

### Data availability.

The genome sequence and associated data for phage Maine were deposited under GenBank accession number MN045228, BioProject accession number PRJNA222858, SRA accession number SRR8869226, and BioSample accession number SAMN11360374.
